# Virtual patients design and its effect on clinical reasoning and student experience: a protocol for a randomised factorial multi-centre study

**DOI:** 10.1186/1472-6920-12-62

**Published:** 2012-08-01

**Authors:** James Bateman, Maggie E Allen, Jane Kidd, Nick Parsons, David Davies

**Affiliations:** 1 Education and Development Research Team, Warwick Medical School, Coventry CV4 7AL, UK; 2 University Hospitals Coventry and Warwickshire, NHS Trust, Lakin Road, Coventry CV2 2DX, UK; 3 Department of Statistics, Warwick Medical School, Coventry, CV4 7AL, UK

**Keywords:** Virtual patients, Clinical reasoning, Elearning, Education, Undergraduate, Musculoskeletal, Rheumatology

## Abstract

**Background:**

Virtual Patients (VPs) are web-based representations of realistic clinical cases. They are proposed as being an optimal method for teaching clinical reasoning skills. International standards exist which define precisely what constitutes a VP. There are multiple design possibilities for VPs, however there is little formal evidence to support individual design features. The purpose of this trial is to explore the effect of two different potentially important design features on clinical reasoning skills and the student experience. These are the branching case pathways (present or absent) and structured clinical reasoning feedback (present or absent).

**Methods/Design:**

This is a multi-centre randomised 2x2 factorial design study evaluating two independent variables of VP design, branching (present or absent), and structured clinical reasoning feedback (present or absent).The study will be carried out in medical student volunteers in one year group from three university medical schools in the United Kingdom, Warwick, Keele and Birmingham. There are four core musculoskeletal topics. Each case can be designed in four different ways, equating to 16 VPs required for the research. Students will be randomised to four groups, completing the four VP topics in the same order, but with each group exposed to a different VP design sequentially. All students will be exposed to the four designs. Primary outcomes are performance for each case design in a standardized fifteen item clinical reasoning assessment, integrated into each VP, which is identical for each topic. Additionally a 15-item self-reported evaluation is completed for each VP, based on a widely used EViP tool. Student patterns of use of the VPs will be recorded.

In one centre, formative clinical and examination performance will be recorded, along with a self reported pre and post-intervention reasoning score, the DTI. Our power calculations indicate a sample size of 112 is required for both primary outcomes.

**Discussion:**

This trial will provide robust evidence to support the effectiveness of different designs of virtual patients, based on student performance and evaluation. The cases and all learning materials will be open access and available on a Creative Commons Attribution-Share-Alike license.

## Background

### Virtual patients

Virtual patients(VPs) can be defined as electronic representations of realistic clinical cases [1]. They have been proposed as being an ideal tool to teach clinical reasoning skills [2]. A recent literature review, and systematic review of the literature has highlighted a lack of evidence supporting individual design properties for virtual patients as in other elearning areas [3]. VPs are widely used in up to one third of US and Canadian medical schools, however until 2007 development costs were high [4]. Multiple tools now exist to author virtual patients Much of the focus until now was been on their utility as educational tools in comparison to traditional teaching, in keeping with other elearning research [5].

A range of software packages exist for case authoring including ‘CAMPUS’, University of Heidelberg [6]; ‘Labyrinth’ from the University of Edinburgh [7]; ‘Web-SP’ from the Karolinska Institute, Sweden [8]; and ‘vpSim’ from the University of Pittsburgh [9]. Other researchers used bespoke software solutions to author cases [10,11].

An international technology interoperability standard for VPs was adopted in 2009 by the Medbiquitous Consortium [12]. This benchmark allows the interoperability of VP cases between compatible software systems allowing authoring, editing and playing of cases [13]. This facilitates collaboration, research, open access and the upkeep of these electronic resources [14,15]. This has potentially changed the working definition of what a VP is, by re-defining properties and dimensions of VPs[16,17]. A European Commission funded study has produced self-reported evaluation scores to help evaluation, the EViP project [15].

There are numerous VP design properties identified in the literature [2] and their impact on the learning experience are poorly understood[3]. Of particular interest are, firstly, the use of branching case pathways [18], and secondly, the role of structured feedback to promote clinical reasoning. Branching cases are more difficult to construct, more expensive when compared with linear cases [4], and may have unpredictable effects on individual students [19]. Clinical reasoning teaching support provides structured approaches to clinical reasoning [20,21], which can be deployed in VPs. In the ‘SNAPPS’ approach [20], students summarise case findings, narrow a differential diagnosis, analyse the differential diagnosis by comparing and contrasting possibilities, and plan management.

A number of validated tools exist to evaluate clinical reasoning and student experiences with a VP. To measure clinical reasoning, tools include the Key Feature Problem [22,23], and the Diagnostic Thinking Inventory[24,25]. Other appropriate assessments include multiple-choice questions, Bayesian reasoning questions [25,26] and diagnostic proficiency [20].

In musculoskeletal medicine there is a challenge in meeting the needs of increasing student populations [27]. This is confounded by a lack of exposure to clinical cases [28] which can potentially be mitigated by the use of virtual patients.

### Problem statement and hypothesis

The influence of different design features in a VP is under-researched, although they can significantly affect the time and cost of VP production. A research study in this area would be able to answer this research question.

We hypothesise that the two important independent VP design variables, branching (present or absent), and structured clinical reasoning feedback (present or absent) are likely to influence students clinical reasoning in cases, and their user experience in terms of realism, engagement and learning value.

## Methods

### Objectives

The aim of this study is to evaluate how two independent VP design variables influence their effectiveness as an educational tool in musculoskeletal medicine. The specific objectives in the study are firstly to evaluate the performance of students exposed to different virtual patient designs in identical assessments of clinical reasoning skills. Secondly we aim to determine how different VP designs influence the student experience when using a VP. Finally we are attempting to explore the relationships between student performance in VP assessment metrics and other measurements of clinical skills, including written and clinical examinations.

### Study design

This is a randomised 2x2 factorial design study evaluating two independent variables of VP design, branching (present or absent), and structured clinical reasoning feedback (present or absent).

### Setting and participants

The setting is three university medical schools in the United Kingdom. These are the Warwick Medical School (WMS), the University of Birmingham Medical School (UBMS), and Keele Medical School (KMS). WMS runs a four year MBChB degree open only to graduate entry medical students, UBMS and KMS have a five year MBChB degree course, open to undergraduate entry medicine (UEM) graduate entry medical (GEM) students. The research project will run from 2011 to 2013.

### Virtual Patient software information technology

Virtual patient cases in the study are created to the Medbiquitous standard [12] using the XML programming language [29]. The software used to create and host the cases is DecisionSim® v2.0, developed by the University of Pittsburgh. The cases are compatible with open source VP systems such as Open Labyrinth [7]. Access to cases, participation, electronic consent, and post case evaluations will be controlled by the VP software, and content hosted on the University of Warwick virtual learning environment Internet pages. Students will be registered with and logged in to the software, allowing tracking of decisions and performance.

### Randomisation

**Figure 1:**
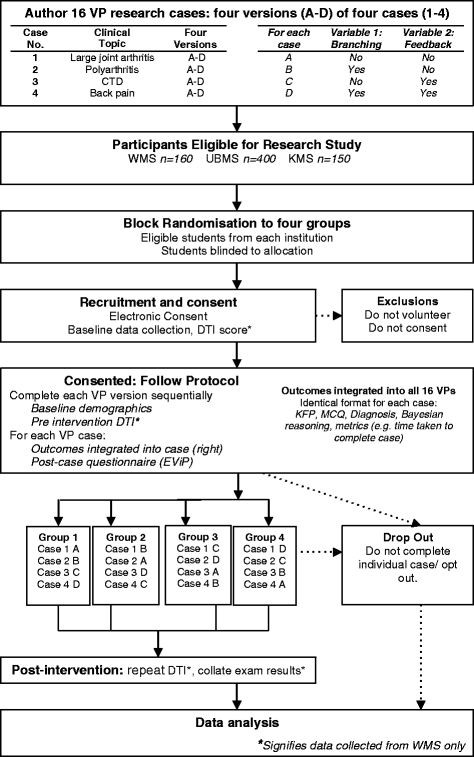
Flow diagram of study Proposed study protocol.

The study follows the CONSORT statement on randomised trials [30]. A flow diagram of the study design is seen in Figure 1. Students from the eligible year-groups in each institution will be allocated to one of four intervention groups using block randomisation. Each of the university cohorts will be randomised individually. Block randomisation will use a computerised random number generator to allocate students. The primary investigator (JB) will implement the allocation and hold a record of the sequence.

### Recruitment and baseline data collection

All eligible students will be invited to participate in the study. Inclusion criteria are students in the year group studying MSK medicine. The only exclusion criteria are students who do not volunteer or consent. Eligible students will be invited to attend an oral presentation and demonstration of the study, and given an approved study participant information sheet. Students who do not electronically record their informed consent will not be able to complete any cases, and are not considered to be participants. Students who consent will be considered to be study participants from this point onwards. At this point the baseline data collected from students will be gender, email address, student type (UEM/ GEM), year of study, and institution.

Additional data and information on other aspects of student performance will be collected from the examinations officer at WMS only. This includes student performance on formative clinical and written assessments at both at the end of the musculoskeletal block, and the end of year assessments.

### Intervention and Independent design variables

The intervention consists of students completing four VP cases sequentially. Each case takes approximately 30 minutes to complete. The cases focus on four core clinical musculoskeletal areas. These are large joint arthritis, back pain, polyarthritis, and connective tissue disease. The 2x2 factorial study design means that any cases can be designed in four different ways. The four case designs are: A) not branched+ no-feedback; B) branched+ no feedback; C) not branched+ feedback; D) branched+ feedback. Students will use all four of the case designs during the research (see Figure 1).

The first variable is branching pathways through the VP, present or absent. There are four branching points with three choices through the thirty-minute case. This gives a possible 81 core pathways (3^4) through the case in a branched form. The linear case has a single core pathway, with participants being redirected back to the core pathway irrespective of the decision made, for example by feedback from a supervising clinician in the case. The second variable is the use of structured feedback to promote clinical reasoning skills, present or absent. This will be in a predetermined approach through the cases at five key points through the case, based on the ‘SNAPPS’ approach [20], systematic approaches to help Bayesian reasoning [21], and symptom categorisation [31].

Cases will be piloted and tested by healthcare professionals and a cohort of students in one centre prior to the study commencing. For the study, students will complete cases at WMS and KMS is in the form of sequential teaching sessions to students, taking place in a computer cluster. Students at UBMS will complete cases during allocated self-study time during their musculoskeletal block.

Other than the described independent variables we will control for other design variables highlighted in a critical literature review [2].

### Inclusion and exclusion criteria

Inclusion criteria are students enrolled on the medical degree course and in the musculoskeletal teaching block in one of the medical schools in the study. Students must electronically sign consent to be included. Exclusion criteria are students from other year-groups. Students registered for a medical degree are required and assumed to have appropriate language and information technology skills.

### Blinding

Students will be blind to their group allocation. Investigator blinding for the purposes of the data analysis and allocation is not used. In the institution where clinical examination performance is recorded, none of the investigators examine within the clinical specialty (musculoskeletal medicine).

### Outcome measures

The primary outcome measures in this study are the performance in standardised composite clinical reasoning assessment using validated tools, and a modified self reported 15-item evaluation, reviewing four domains. These will be completed both during and immediately following each case. The secondary outcome measures are engagement and patterns of use within the cases and collected from the online environment (see Table 1). For each case, the composite clinical reasoning assessment consists of validated assessment of clinical reasoning. These 15 items are eight ‘key feature problem’ questions, one Bayesian reasoning question, two multiple choice questions on diagnosis, and four multiple choice clinical decision questions. For each case the content of these 15 items is identical, allowing comparison between case designs. This allows comparison of a case which is not branched with no structured feedback vs. the same case in a branched format with structured feedback.

**Table 1:** Outcomes Measured during the study

***Institution***	***Outcomes for individual cases***	***Timing***
**All institions (WMS, KMS, UBMS)**		
**Primary Outcome Measures collected for each VP**		
*Validated clinical reasoning assessments.*	Key Feature Problems score (x/8)	Student completes during the case
	Clinical Decision (x/4)	
	Bayesian Statistical Question (x/1)	
	Working diagnosis (x/2)	
	(Total score x/15)	
*Self reported evaluation (EVIP)*	*EViP Questionnaire (multiple domains)*	After each case
	*Case Preference: reasoning (n from 4) learning (n from 4)*	On completion of all cases
	*Preference of case (learning)*	
	*Preference of case (realism)*	
**Secondary Outcome measures**		
*Other metrics collected in electronic environment*	Time spent per case (seconds)	During the case, recorded automatically
	Time spent per node (seconds)	
	Number of nodes visited	
	Case completion percentage.	
	Time spent per question (seconds)	
**WMS Only**		
*Validated self reported reasoning assessment (DTI)*	Baseline and Mean improvement	Immediately Pre-and post- intervention
*Summative assessment*	Written and clinical	1 week post intervention
*Formative assessment*	Written and clinical	End of the year

Student evaluation of each case will be collected using an electronic version of the EViP questionnaire, a fifteen item self reported evaluation. This explores exploring authenticity, professionalism, learning, and coaching through the case, using Likert scales with additional free text responses. Secondary outcome measures for each case are student’s patterns of use of the case, such as time taken per case, case completion rates, and time taken to complete individual decisions.

Additional data will be collected from one centre, WMS, to support the validity of the VPs as educational and assessment tools. This includes a pre- and post-test Diagnostic Thinking Inventory, a 41 item validated assessment of clinical reasoning ability [24]. Performance in summative and formative written and clinical assessments, measured one week following the VP case, and several months later will also be collected.

### Sample size determination

The authors agreed an important educational effect of a 5% difference in the score on validated assessments of clinical reasoning skills, and student self reported evaluations score. As no gold standard exists for the measurement of clinical reasoning skills, we have based the sample size calculation on performance for clinical reasoning on performance in the key feature problems (KFPs) integrated into each VP case. A previous study has shown mean KFP scores in a student population to be approximately normally distributed, with a standard deviation of 1.32 [32]. In this study where we will use 16 KFPs, a 5% difference in scores is considered significant, that is a difference in mean scores of 0.8, corresponding to a standardised effect size of approximately 0.6 (moderate to large). Based on these assumptions, we would require a total sample size of 88 students to detect this difference with 80% power at the (two-sided) 5% level. Assuming the effect size to be the same for both branching and feedback interventions, a sample size of 88 students would provide sufficient power to detect the main effects and an interaction effect that was twice as large as the assumed main intervention effect in the setting shown in Table 2.

**Table 2:** Sample size calculation for Key Feature Problems outcomes, and student self evaluation scores

**Key Feature problems**					**Student self reported Evaluation scores**				
		Branching		*Total*			Branching		Total
		No	Yes				No	Yes	
Feedback	No	22	22	*44*	Feedback	No	28	28	*56*
	Yes	22	22	*44*		Yes	28	28	*56*
*Total*		*44*	*44*	***88***	*Total*		56	*56*	***112***

If the interaction between branching and feedback interventions is of same order of magnitude as the expected main effects then we would require a fourfold increase in the sample size to give a total of 352 students. [33]

For self-reported scores, where a previous study reported a standard deviation of 0.93, [34] a 10% difference in scores (with a maximum of 5) is considered significant, that is a difference in mean scores of 0.5, corresponding to a standardised effect size of approximately 0.5 (moderate). Based on these assumptions, we would require a total sample size of 112 students to detect this difference with 80% power at the (two-sided) 5% level (Table 2).

Therefore 112 students would be required to detect the main effects and large interaction effect of branching and feedback on self-reported scores. To detect an interaction effect of the same order of magnitude as the expected main effects would require a total of 448 students. The pool of students available for recruitment into this study is large at the three centres (WMS, n~160; UBMS, n~400; KMS, n~150). Given unforeseen recruitment problems and some loss to follow-up, a target of 112 students should be easily achievable to quantify the main intervention effects (branching and feedback) which are the primary focus of the study, with increasing recruitment above this target providing increasing power to detect potential interactions between the main effects.

### Data analysis

We will present absolute numbers for enrolment, eligibility, and complete follow up. Descriptive statistics will be used to present student demographics, along with the mean, standard deviation, standard error of the mean, and 95% confidence intervals for primary and secondary outcome measures.

The primary analysis will be based on complete cases on a per-protocol analysis. It seems likely that some data may not be available due to voluntary withdrawal of participants, or drop-out through lack of completion of individual data items , unforeseen technical difficulties, and general loss to follow-up. Where possible the reasons for data ‘missingness’ will be ascertained and reported. The pattern of the missingness will be carefully considered and the reasons for non-compliance, withdrawal or other protocol violations will be stated and any patterns summarised. The primary analysis will investigate the fixed effects of the factorial combinations of branching and feedback on the primary outcome measures, performance in a standardised composite clinical reasoning assessment and a 15-item self reported evaluation. Analysis of covariance (ANCOVA) will be used to identify main effects, effect sizes, and interactions between the two independent design variables (feedback and branching). Blocking factors in the ANCOVA will adjust for the effects randomisation group, case ordering and recruiting centre, with student GEM status and gender as covariates. Tests from the ANCOVA will be two-sided and considered to provide evidence for a significant difference if p-values are less than 0.05 (5% significance level). Estimates of treatment effects will be presented with 95% confidence intervals. Students case preferences for learning and realism, and EViP will be evaluated using chi-squared tests for grouping factors case design and number.

We will determine the predictive validity of performance in the VP composite assessment, using one institution’s summative examination results, WMS. We will use the correlation coefficient (Pearson’s product–moment, *r*) to determine the effect size of any linear correlation between the VP scores and institution examinations.

A detailed statistical analysis plan (SAP) will be agreed with the trial management group at the start of the study, with any subsequent amendments to this initial SAP being clearly stated and justified. The routine statistical analysis will mainly be carried out using R (http://www.r-project.org/) and S-PLUS (http://www.insightful.com/). Results from this study will also be compared with results from other studies.

## Ethical approval

The National Health Service Local Research Ethics Committee approved the protocol as an educational research study. Warwick Medical School Biomedical Research Ethics Committee gave written ethics and institution approval in 2010. The study has institutional approval from KMS and UBMS.

## Discussion

The main purpose of this randomised-factorial study design is to identify the most effective design principles for VPs across a range of musculoskeletal cases, addressing a research question identified recently in the literature [2], which have not been answered by a recent meta-analysis [3].

Our use of validated assessments of clinical reasoning where possible helps to validate the research findings, however there are limitations in these existing tools to measure clinical reasoning. The use of assessment data from one institution from both summative and written examinations will assist with the interpretation of the predictive and criterion validity of the VP cases when considered as formative assessments.

The blinding of students to group allocations will hopefully minimise bias and preconceptions about virtual patients. The students in the study do not have VP education formally integrated into any curriculum, however previous exposure and familiarity with existing open access cases cannot be excluded.

Interpretation of the research findings will be facilitated by open access publication of VPs generated. This has not been used in recent published peer reviewed research on VPs [2,3,34-36]. The publication of these cases with the research will allow appropriate integration of the materials as a learning resource of the design process, and allow other researchers to evaluate the research methods [37].

## Competing interests

The authors’ declare that they have no competing interests.

## Authors’ contributions

All of the authors have had a substantial contribution to the research design and study protocol. JB and DD conceived the original study design, which was reviewed by MA and JK. NP advised on the statistical analysis and power calculations. JB wrote the initial draft, which was revised and approved by MA, JK, NP and DD for content. All authors have approved the final draft of this work for publication.

## Authors’ information

JB is an Education Research Fellow with Arthritis Research UK, undertaking a PhD at Warwick Medical School. MA is a consultant rheumatologist and Director of Medical Education at University Hospitals Coventry and Warwickshire NHS Trust, where NP works as a statistician with Warwick Medical School. JK is head of the Education Development and Research Team (EDRT), Warwick Medical School. DD is Associate Professor of Medical Education at the EDRT.

## Pre-publication history

The pre-publication history for this paper can be accessed here:

http://www.biomedcentral.com/1472-6920/12/62/prepub
